# Comprehensive analysis of mitophagy in HPV-related head and neck squamous cell carcinoma

**DOI:** 10.1038/s41598-023-34698-4

**Published:** 2023-05-09

**Authors:** Li Yanan, Liang Hui, Cheng Zhuo, Ding Longqing, Sun Ran

**Affiliations:** 1 Department of Otorhinolaryngology, Shandong First Medical University & Shandong Academy of Medical Sciences, Ji’nan, 250000 Shandong China; 2 Department of Otorhinolaryngology, The First Affiliated Hospital of Shandong First Medical University & Shandong Provincial Qianfoshan Hospital, Ji’nan, 250014 Shandong China

**Keywords:** Cancer, Diseases, Oncology

## Abstract

Head and neck squamous cell carcinoma (HNSCC) is a common tumour type in otorhinolaryngology, and its occurrence is related to long-term exposure to tobacco and alcohol. Recently, HPV infection has become an increasingly important contributor to HNSCC, and HPV-associated HNSCC has a different clinical course and better prognosis than non-HPV-associated HNSCC. However, the exact molecular mechanism of HNSCC is unclear. Here, we obtained data from The Cancer Genome Atlas (TCGA) and gene expression omnibus (GEO) to analyse the mitophagy process and related influencing factors of HPV-associated HNSCC via the integration of bioinformatics analysis and experimental validation. We found that in HPV-associated HNSCC, process of mitophagy affects tumour development, immune cell infiltration and prognosis. In the mitophagy process of HPV-related HNSCC: NOS2, IL17REL, TMSB15A, TUBB4A and other hub genes showed significantly higher expression levels than in non-HPV-related HNSCC. Furthermore, this was also confirmed by quantitative real-time PCR (qRT‒PCR), which was used to detect the expression of differentially expressed genes in HNSCC tissues. Furthermore, we found that the unique immunological characteristics by expressing CD8^+^ T cell in a high level in HPV-related HNSCC, and the scores obtained from the score model affected the prognosis of patients. In conclusion, our study revealed the unique biomolecular signature of mitophagy in HPV-associated HNSCC, which may contribute to the development of precise treatment regimens for HPV-associated HNSCC patients.

## Introduction

Head and neck squamous cell carcinoma (HNSCC) is generally associated with tobacco consumption, alcohol abuse or both. However, it has recently been increasingly attributed to infection with human papillomavirus (HPV)^1^. HPV is a circular double-stranded DNA, and more than 200 genotypes have been discovered, all of which are divided into high- and low-risk HPV^2^. It is currently believed that the occurrence of HNSCC is mainly related to high-risk HPV infection, especially HPV-16. Persistent viral infection affects the cell cycle and causes carcinoma through the expression of oncoproteins^3^. The survival of patients with HPV-positive head and neck cancer is significantly higher than that of patients with HPV-negative cancer^4,5^, and different studies have shown that change is associated with multiple factors^6–9^.

Mitochondria are important organelles in eukaryotic organisms. Normal mitochondria play an important role in ATP synthesis, calcium buffering, immune monitoring and other processes. When mitochondria are damaged, they can be removed by mitophagy to maintain mitochondrial quality control and mitochondrial quantity^10^. The mechanism of mitophagy in carcinoma cells is significantly more complex than that in normal cells. In colorectal cancer studies, inhibition of mitophagy enhanced the sensitivity of tumour cells to chemotherapeutic drugs^11^. However, inhibition of mitophagy may also contribute to bone metastasis of breast cancer^12^. Maybe, mitophagy plays different roles in the occurrence and development of tumours, and the relationship between mitophagy and HPV-associated HNSCC has rarely been studied; the main research is on the effect of the motiphagy of combination of antitumour drugs on HNSCC^13,14^. We speculate that infection with high-risk HPV alters the process of mitophagy, and the overall prognosis of HPV-associated HNSCC is better than that of non-HPV-associated HNSCC.

To investigate the relationship between mitophagy and HPV-related HNSCC, we determined the differentially expressed genes related to mitophagy and the effects of the mitophagy score on the prognosis and immune cell infiltration by obtaining HPV-related and non-HPV-related HNSCC human specimen datasets from the Cancer Genome Atlas (TCGA) and Gene Expression Omnibus (GEO) databases. To obtain the relationship between mitophagy and HPV-related HNSCC, differential gene screening was performed on HPV-related and non-HPV-related HNSCC samples obtained from TCGA database, and confirmed the differential genes by basic experiment. Then autophagy-related differential pathways were obtained according to differential gene analysis. Then, we combined TCGA and GEO database HPV-related HNSCC samples to construct the mitophagy score model and analyse the effect on immune cell infiltration and prognosis. We found differential gene expression and molecular pathways between HPV-associated and non-HPV-associated HNSCC samples during mitophagy. Furthermore, the infiltration of CD4-positive memory T cells and M2 macrophages was reduced in the group with a higher mitophagy score, and the mitophagy score was more accurate in predicting long-term prognosis.

## Materials and methods

### Data and differentially expressed genes were obtained

HNSCC gene expression profile data were obtained from the TCGA database using the R package TCGA biolinks package (version 2.22.4)^15^, and TCGA-HNSCC HPV-related phenotype information was obtained by Genomic Data Commons (GDC) software. HPV-negative and HPV-positive tissues were distinguished according to p16 test results. After the invalid samples were removed, the remaining samples were classified as 72 HPV-negative tissues and 31 HPV-positive tissues for gene expression analysis. Gene set enrichment analysis (GSEC) was used to identify the mitophagy regulatory gene set GO: 1901524, and 12 functional mitophagy regulatory genes were selected for expression analysis in HNSCC data and to build a mitophagy score. The HNSCC-related dataset GSE65858^16^ was obtained from the GEO database by the GEOquery package^17^, and 196 HPV-negative controls with p16-negative samples and 60 HPV-positive samples with p16-positive samples were identified; all were human samples.

The tumour samples from the HPV-negative group and HPV-positive group in the TCGA database were analysed by the R package rgl package (version 0.107.10) for dimension reduction. Then, the R package ggplot2 package (version 3.3.5) was used to determine the expression differences in mitophagy-regulating genes. Tissue samples from the TCGA database were redivided into 70 A and 33 B types according to mitophagy regulatory genes. The R package DESeq2(version 1.34.0)^18^ was used for differential analysis based on the new grouping, and 76 upregulated differentially expressed genes(DEGs) and 321 downregulated DEGs were finally obtained. We set the genes with logFC > 1 and adj*P* value < 0.05 as upregulated differentially expressed genes and the genes with logFC < − 1 and adj*P* value < 0.05 as downregulated differentially expressed genes. Mitophagy regulation and DEGs were displayed in heatmaps by the pheatmap package.

### Quantitative reverse transcription-PCR

We selected 6 cases of human HPV-negative HNSCC tissue and 6 cases of human normal tonsil tissue (all patients were selected from The First Affiliated Hospital of Shandong First Medical University & Shandong Provincial Qianfoshan Hospital) for random combination. Each case of HNSCC tissue corresponding to a case of normal tonsil tissue was divided into 6 groups for control experiment analysis of some differentially expressed genes in mitophagy. The same experimental procedure was performed for each group: the rice grain-sized tissues were removed from liquid nitrogen and ground into powder by a grinder. RNA extraction was performed using TRIzol (Vazyme, Nanjing, Jiangsu Province, China), and the RNA was measured with a 2000 spectrophotometer. The concentration and purity of the extracted RNA were determined(Thermo Fisher Scientific, USA) according to the instructions of the HiScript III RT SuperMix for qPCR kit. The extracted RNA was reverse transcribed into cDNA, and 2 × ChamQ Universal SYBR qPCR Master Mix reagent was used for PCR on the StepOnePlus PCR system (Thermo Fisher Scientific, USA). The following steps were performed under the following conditions: first, predenaturation at 95 °C for 30 s, then 95 °C for 10 s, 60 °C for 30 s, and 95 °C for 15 s, 60 °C for 60 s, 95 °C for 15 s, for a total of 40 cycles. The primer sequences for PCR amplification were as follows: IL17REL, forward: 5′-GCTGTGGGACACGGTCTACTA-3′, reverse:5′-GCTGGATTGCTGCAGCTTACG-3′; NOS2, forward: 5′-CCTGGCAAGCCCAAGGTCTA-3′, reverse: 5′-CGCACATCCCCGCAAACATAG-3′; TMSB15A, forward:5′-CCTCCCAACAGCAGATTTCGAC-3′, reverse: 5′-TCCGAAGACGCCTAAAATCTCTACA-3′; TUBB4A, forward:5′-TCGATGCCAAGAACATGATGGC-3′, reverse: 5′-TGTTCTTGCTCTGCACGCTCA-3′; GAPDH, forward: 5′-GCACCGTCAAGGCTGAGAAC-3′, reverse:5′-TGGTGAAGACGCCCAGTGGA-3′. The relative mRNA expression level was calculated by the 2–11 Ct method and standardized to GAPDH.

### Enrichment analysis

Cluster Profiler packages (version 4.2.2)^19^ were used for Gene Ontology(GO) annotation analysis and Kyoto Encyclopedia of Genes and Genomes (KEGG)^20–22^ pathway enrichment analysis of differentially expressed genes, and a critical value of FDR less than 0.05 was considered to be statistically significant. To differentiate the biological processes of the two groups, according to the gene expression profile database of TCGA HNSCC samples, gene set enrichment analysis (GSEO)^23^ was used for gene set enrichment analysis. "c2.cp.kegg.v6.2. -symbols" gene set was obtained from the MSigDB database^24^ for GSEA. A false discovery rate (FDR) < 0.25 was considered to be significantly enriched. Using the R package GSVA (gene set variation analysis)^25^, single-sample gene set enrichment analysis (ssGSEA) was used to calculate the scores of related pathways based on the gene expression matrix of each sample, and differences in enrichment functions (or pathways) were screened by the limma package(version 3.50.1)^26^.

### Construction of the mitophagy regulatory scoring model

The glmnet packet (version 4.1.3)^27^ was used to construct the scoring model with the differential genes obtained by typing, and the parameters were set as follows: seed (2), family = "binomial". Based on the screened TCGA-HNSCC and GSE65858 datasets, univariate Cox regression analysis and LASSO regression analysis were used to further screen the prognostic genes and then establish the prognostic model. First, we used univariate Cox regression analysis to calculate the association between the expression of each differential gene and OS (overall survival) and retained the genes with a *P* value < 0.05. Then, the combined dataset was randomly divided into a training set and validation set, and the Lasso algorithm was used to eliminate multicollinearity in the training set and select the meaningful variables in univariate Cox regression analysis. Finally, the risk score formula was calculated by considering optimized gene expression and the correlation estimation Cox regression coefficient: ${\text{risk}}\;{\text{score}}\,{ = }\,\left( {{\text{DMBX}}1*0.118814319} \right) + \left( {{\text{KRT}}2* - 0.000610081} \right) + \left( {{\text{NPPC*}}0.004718129} \right)$. According to the given score, the patients were divided into a high-score group and a low-score group. Kaplan–Meier analysis and the log-rank test were performed using the survival packet (version 3.2.13) to analyse the overall survival of the validation set. In addition, we used time-dependent receiver operating characteristic (ROC) curves to assess survival predictions and the time ROC R package (version 0.4)^28^ to calculate area under the ROC curve values to measure prognosis or prediction accuracy. Validation sets were tested using scoring formulas to assess survival prediction and prediction accuracy.

### Effect of the mitophagy score on immune infiltration

We uploaded the expression matrix data of the training set and validation set to CIBERSORTx, combined with the LM22 characteristic gene matrix, filtered the output samples with *P* < 0.05, and obtained the immune cell infiltration matrix. A histogram was drawn using the R language ggplot2 package to show the distribution of 22 immune cell infiltrates in each sample.

### Statistical analysis

To compare two sets of continuous variables, the independent Student’s t test was used to estimate the statistical significance of normally distributed variables, and the Mann‒Whitney U test (or Wilcoxon rank sum test) was used to analyse the differences between nonnormally distributed variables. The receiver operating characteristic curve was plotted using the R time ROC package, and the area under the curve (AUC) was calculated to assess the accuracy of the risk score in estimating prognosis. All statistical *P* values were bilateral, and *P* < 0.05 was considered statistically significant.

### Ethics approval and consent participate

The study was performed in accordance with the ethical standards of the Declaration of Helsinki (1964) and its subsequent amendments. All experimental protocols were approved by the Medical Ethics Committee of the First Affiliated Hospital of Shandong First Medical University & Shandong Provincial Qianfoshan Hospital. All participants provided written informed consent before participating, all minors have the consent of their guardians and the guardian signed the informed consent.

## Results

### The difference and mutations in mitophagy regulate gene expression

**Figure 1:**
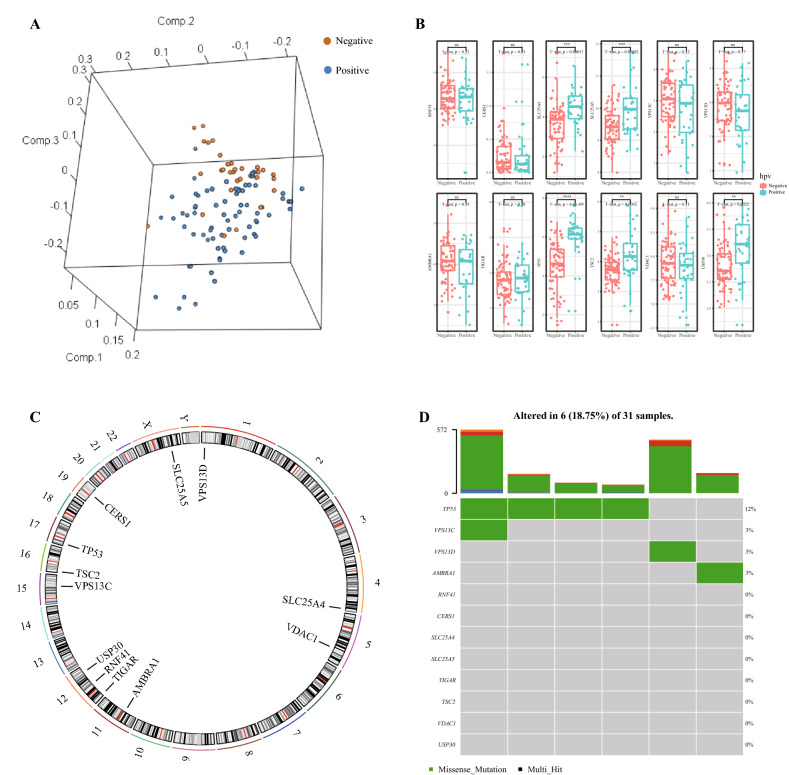
(**A**) Differences and mutations in mitophagy regulate gene expression. PCA was plotted according to groups of HPV-positive and HPV-negative. (Negative-HPV-negative, Positive-HPV-positive). (**B**) The box diagram shows the expression of mitophagy regulatory genes in HPV-negative and HPV-positive samples. (**C**) The localization of mitophagy regulatory genes on chromosomes. D.Mutation of mitophagy regulatory genes in HPV-positive HNSCC. In (**D**), rows represent the number of patients with HNSCC, and the columns represent different genes.

Seventy-two HPV-negative tissue samples and 31 HPV-positive tissue samples obtained from the TCGA-HNSCC dataset were subjected to principal component analysis (PCA) dimensionality reduction. The results showed that HPV-grouped samples could be clearly distinguished (Fig. 1A). The expression of mitophagy-regulating genes (Fig. 1B) and their chromosomal localization (Fig. 1C) were analysed according to HPV grouping. The results showed that the SLC25A4, SLC25A5, TP53, TSC2 and USP30 genes were highly expressed in HPV-positive HNSCC samples.

**Figure 2:**
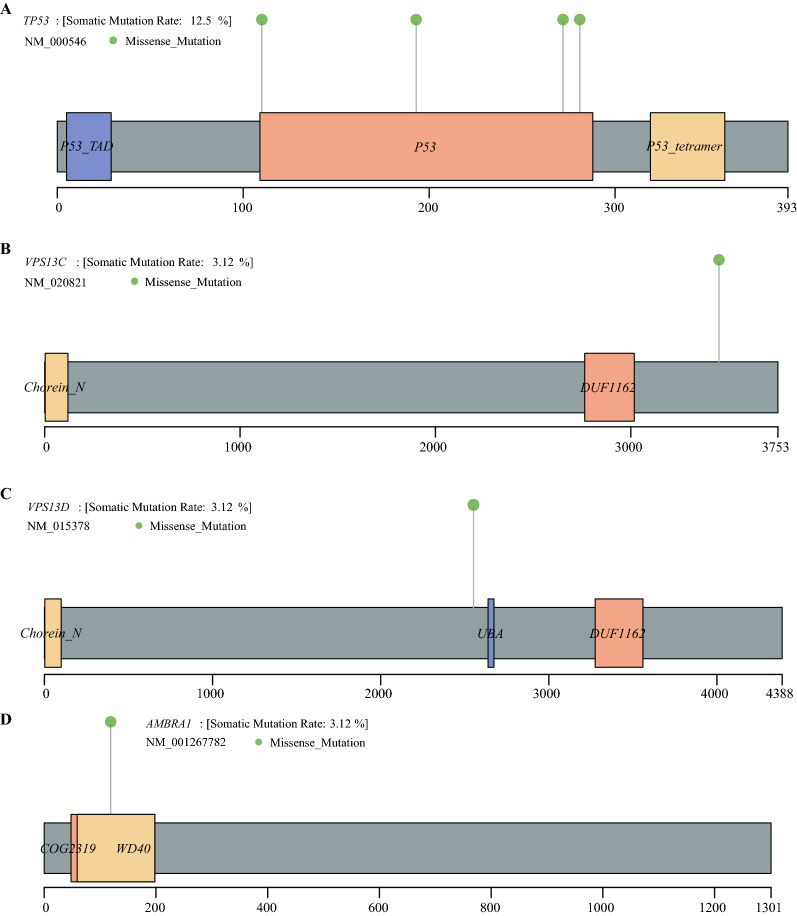
Mutations in mitophagy regulatory genes. Possible mutation points of TP53 (**A**), VPS13C (**B**), VPS13D (**C**) and AMBRA1 (**D**).

Then, mutations in mitophagy regulatory genes in 31 HPV-positive samples were analysed. The waterfall map showed that TP53, VPS13C, VPS13D and AMBRA1 may have mutations in HPV-related HNSCC (Fig. 1D). Furthermore, a lollipop map was drawn to show the possible mutation sites of the above four genes. The results showed that there are 4 mutation points in TP53 (Fig. 2A) and 1 mutation point in VPS13C, VPS13D and AMBRA1 (Fig. 2B–D).

### Verification of NOS2, IL17REL, TMSB15A, and TUBB4A downregulated expression between non-HPV-related HNSCC and normal tonsil tissue by qRT‒PCR

**Figure 3:**
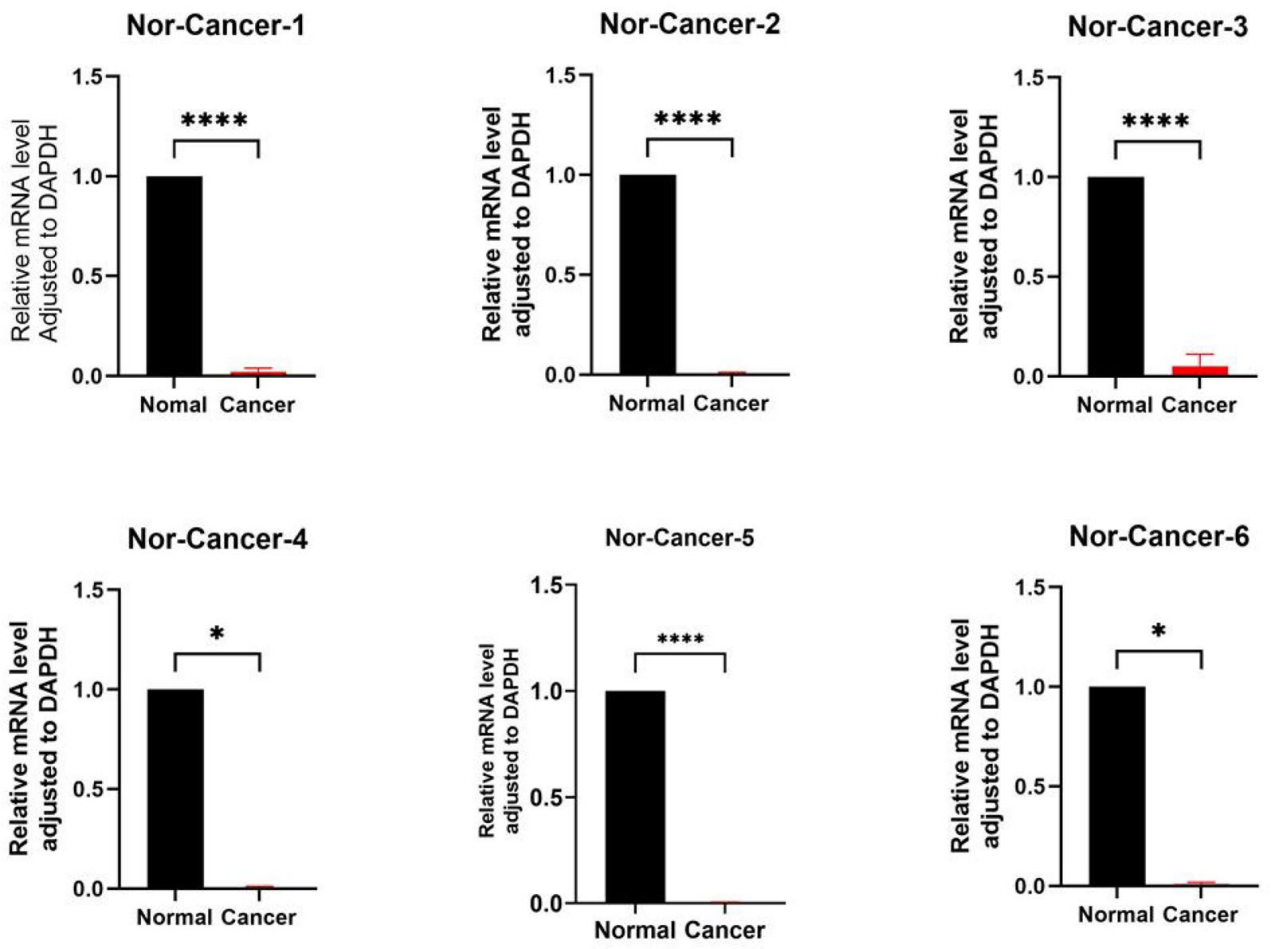
To determine the mRNA expression of NOS2, IL17REL, TMSB15A, and TUBB4A in non-HPV-related HNSCC and normal tonsil tissue, we performed qRT‒PCR analysis. Asterisks indicate the magnitude of the P value, and one asterisk indicates a *P* value < 0.05.

To verify the difference in NOS2, IL17REL, TMSB15A, and TUBB4A expression between non-HPV-related HNSCC and normal tonsil tissue, we performed qRT‒PCR to measure the expression of NOS2, IL17REL, TMSB15A, and TUBB4A at the transcriptional level and found that NOS2, IL17REL, TMSB15A, and TUBB4A expression in non-HPV-related HNSCC tissues was significantly lower than that in normal head and neck tissues (*P* value < 0.05, Fig. 3).

### Analysis of mitophagy regulatory gene subtypes

**Figure 4:**
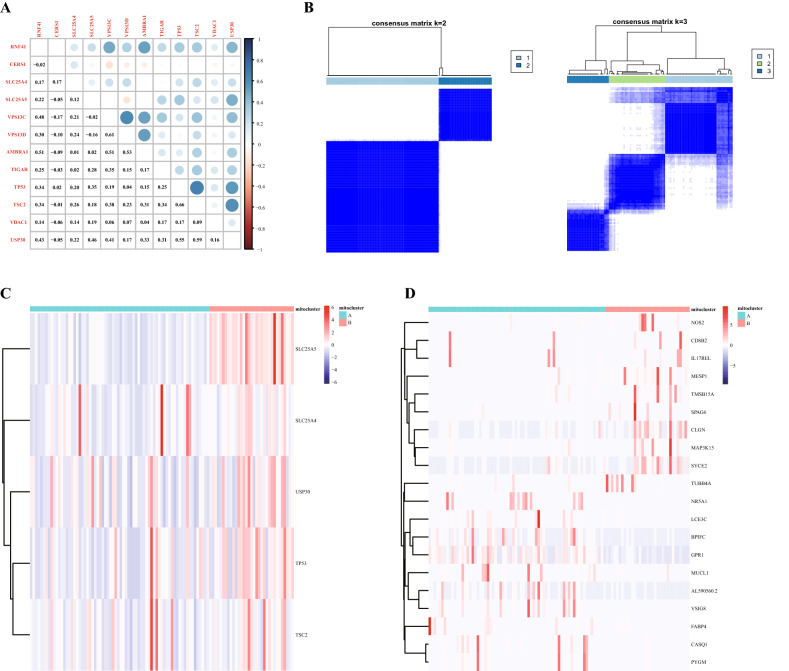
Analysis of mitophagy regulatory gene subtypes. (**A**) Correlation analysis of mitophagy regulatory genes. (Software: R (4.0.2) version. R packet: ggplot2 [3.4.2]. URL: https://ggplot2.tidyverse.org; https://github.com/tidyverse/ggplot2). (**B**) Consistency clustering analysis of the dataset. Heatmap based on subgroups of mitophagy regulatory genes. (Software: R (4.0.2) version. R packet: Consensus Cluster Plus [1.62.0]. URL: https://bioconductor.org/packages/ConsensusClusterPlus/; https://code.bioconductor.org/browse/ConsensusClusterPlus/). (**C**) High expression of mitophagy regulatory genes in HPV-positive samples is shown. (**D**) Heatmap based on subgroups of mitophagy regulatory genes showing the top 20 differentially expressed genes. In the heatmap, red is active, and blue is inhibited. (**C**–**D**) Software: R (4.0.2) version. R packet: heat maps [1.22.0]. URL: https://bioconductor.org/packages/heatmaps/; https://code.bioconductor.org/browse/heatmaps/).

We determined the 12 gene correlations of each mitophagy regulatory gene set (Fig. 4A), and the results showed that these genes had good correlations. The "Consensus Cluster Plus" R package was used for consistent clustering of HPV-related datasets in TCGA-HNSCC. When the typing number parameter was 2, the samples could be classified well (Fig. 4B). Then, we used the model genes obtained to draw a heatmap by grouping subtypes of mitophagy regulatory genes, and the results showed the trend of GEDs. Subtype A was related to low expression of mitophagy regulatory genes, and subtype B was related to high expression of mitophagy regulatory genes (Fig. 4C). The DESeq2 package was used for differential analysis of sequencing data to obtain the differentially expressed genes of the two groups of data, and the differentially expressed genes were screened. The genes with logFC > 1 and adj*P* value < 0.05 were upregulated differentially expressed genes. The genes with logFC < − 1 and adj*P* value < 0.05 indicated downregulated differential genes with downregulated expression. Finally, 76 upregulated DEGs and 321 downregulated DEGs were obtained, and the heatmap shows the top 20 DEGs (Fig. 4D). The results were stored for subsequent enrichment analysis.

### Enrichment analysis

**Figure 5:**
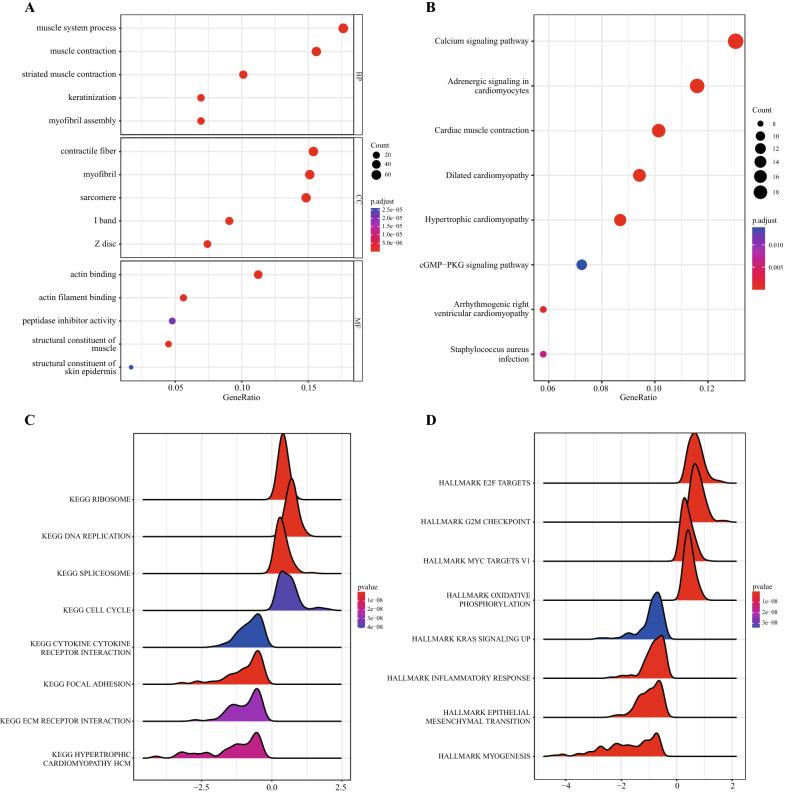
Enrichment analysis. (**A**–**B**) GO functional enrichment analysis. The abscissa is the gene ratio, the ordinate is GO terms, P value the node size represents the number of genes enriched in the pathway, and the node colour represents − log10. BP, biological process. MF, molecular function. CC, cellular component. (**C**–**D**) KEGG pathway enrichment analysis: the horizontal coordinate is the gene ratio, the vertical coordinate is the pathway name, the node size represents the number of genes enriched in the pathway, and the node colour represents − log10 (P value). GSEA of mitophagy regulatory gene subtypes was performed, and the results were visualized in the form of a mountain map. The abscissa is the gene ratio, the ordinate is the KEGG pathway, and the colour represents the P value. GSEA of mitophagy regulatory gene subtypes was performed, and the results were visualized in the form of a mountain map. The abscissa is the gene ratio, the ordinate is HALLMARK, and the colour represents the P value. (Software: R (4.0.2) version. R packet: cluster Profiler [4.6.2]. URL: https://code.bioconductor.org/browse/clusterProfiler/; https://bioconductor.org/packages/clusterProfiler/).

To analyse the relationship between the 391 DEGs and biological processes, molecular functions, cellular components, biological pathways and diseases, we first performed functional enrichment analysis on the differentially expressed genes. The results showed that the DEGs involved in mitophagy regulation were mainly enriched in muscle system processes, muscle contraction, keratinization, striated muscle contraction, myofibril assembly and other biological processes. Moreover, they were enriched in sarcomere, contractile fibre, myofibril, I Band, Z disc, cornified envelope and other cell components, as well as in structural constituent of muscle, actin binding, actin filament binding, peptidase inhibitor activity, structural constituent of skin epidermis and other molecular functions (Fig. 5A). Then, pathway enrichment analysis of DEGs was performed, and the results showed that the DEGs were enriched in cardiac muscle contraction, adrenergic signalling in cardiomyocytes, dilated cardiomyopathy, hypertrophic cardiomyopathy, calcium signalling pathway and other biological pathways (Fig. 5B).

To determine the effect of gene expression levels on HPV-related HNSCC, we analysed the relationship between gene expression levels and the biological processes involved, the cell components affected, and the molecular functions performed in the two sets of data. The results showed that prognostic differential genes mainly affected KEGG-ribosome, KEGG-focal adhesion, KEG-spliceosome, KEGG-DNA replication, KEGG-hypertrophic cardiomyopathy HCM and other pathways (Fig. 5C) and HALLMARK-E2F targets, HALLMARK-epithelial mesenchymal transition, HALLMARK-G2M checkpoint, HALLMARK-MYC targets V1, HALLMARK-myogenesis and other related functions (Fig. 5D). (Supplementary Table 4 showns the details).

### GSVA

**Figure 6:**
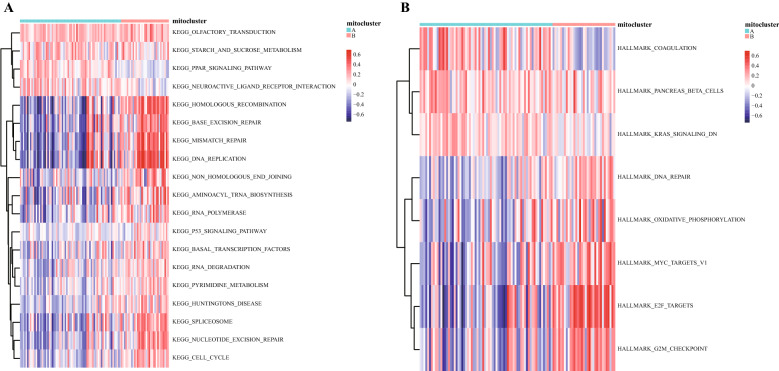
GSVA analysis. (**A**) GSEA-KEGG enrichment results were analysed by GSVA and visualized by heatmap. (**B**) GSEA-HALLMARK enrichment results were analysed by GSVA and visualized by heatmap. Red is active, and blue is inhibition. GSEA, Gene Set Enrichment Analysis. GSVA, gene set variation analysis. KEGG, Kyoto Encyclopedia of Genes and Genomes. HPV, human papilloma virus. HNSC, Head and neck squamous cell carcinoma. (Software: R (4.0.2) version. R packet: heat maps [1.22.0]. URL: https://bioconductor.org/packages/heatmaps/; https://code.bioconductor.org/browse/heatmaps/).

To test the results of gene set enrichment, we conducted GSVA (Gene Set Variation Analysis) to transform the expression matrix of genes between different samples into the expression matrix of genes between different samples to evaluate whether different metabolic pathways were enriched between different samples. Then, the pheatmap package was used to visualize the results (Fig. 6A–B). The results showed that sample grouping could distinguish the results of gene set enrichment analysis. (Supplementary Table 5–6 shows the details).

### Construction of the mitophagy regulatory scoring model

**Figure 7:**
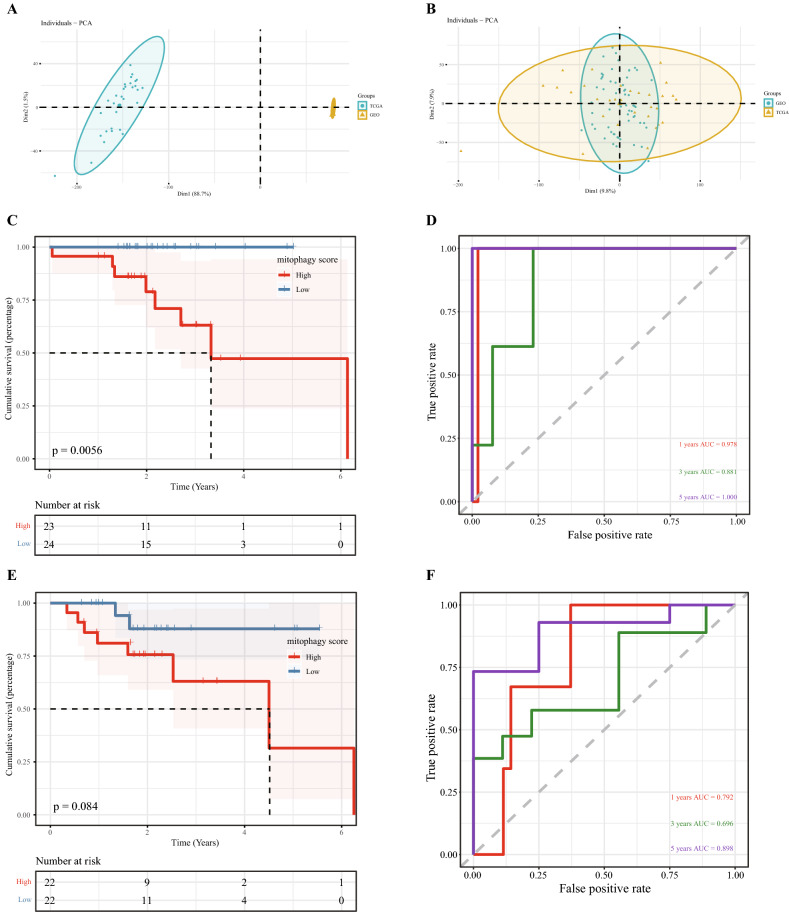
Construction of the mitophagy regulatory scoring model. (**A**) Before data merging, a PCA graph was drawn according to the data source grouping. (**B**) After data merging, PCA was performed according to the data source grouping. The X-axis and Y-axis represent principal component 1 (PC1) and principal component 2 (PC2), respectively. PC1 in the figure (X-axis) can reflect the feature differences in TCGA data, while PC2 in the figure (Y-axis) can reflect the feature differences in GEO data, so the whole PCA figure can reflect nearly half of the data differences. Because the data are high-dimensional, the first two principal components may not reflect the majority of the difference. Thus, the data can be analysed on a case-by-case basis. Each point in the graph represents each sample in the principal component 1 and 2 principal component location information, and the corresponding map of a single sample value cannot reflect the characteristics of the entire sample set. The distance between points (sample) can reflect the difference between samples. Different colours represent different samples belonging to a group, this part from comments head of uploading data content, concrete data format. The two figures show the results for two groups of samples, increasing the confidence of the results (if the differences between groups are too large, there may be no overlapping data points). (**C**) A global survival curve based on the mitophagy regulatory score in a training set. (**D**) ROC curve based on the mitophagy regulatory score in the training set. (**E**) A global survival curve based on the mitophagy regulatory score in a validation set. F.ROC curve based on the mitophagy regulatory score in the validation set.

To increase the reliability of the mitophagy regulatory scoring model, 31 HPV-positive TCGA-HNSCC data were combined with 60 HPV-positive GSE data, and the SVA package was used for batch removal. The PCA dimension reduction cluster diagram before and after batch removal is shown (Fig. 7A–B). The results showed that the data mixed well after the batch effect was removed. Univariate Cox regression analysis was performed on the combined data to calculate the association between the expression of each differential gene and OS, and the genes with a *P* value < 0.1 were retained. Then, the combined dataset was randomly divided into a training set and a validation set. The Lasso algorithm was used to eliminate multicollinearity in the training set and select meaningful variables in univariate Cox regression analysis.Finally, the risk score formula was calculated by considering optimized gene expression and correlation estimation Cox. According to the given score, the patients in the training set and validation set were divided into a high-score group and a group, respectively.

Kaplan‒Meier analysis and logarithmic rank test were performed using the survival package, and the results showed that the overall survival rate of the high-rated and low-rated samples in the training concentration could be well distinguished (Fig. 7C), and the long-term survival rate of the high-rated and low-rated samples in the training concentration could also be well distinguished (Fig. 7E). These findings indicated that the model is more accurate in the assessment of long-term prognosis. In addition, we used time-dependent receiver operating characteristic curves to assess survival predictions and timeROC R packages to calculate area under the ROC curve values to measure prognosis or prediction accuracy. The results showed that the long-term prediction AUC values of both the training set and the validation set were good (Fig. 7D–F). The results of the validation set showed that the model had good efficiency. The AUC of the 1-year prediction was 0.792, the 3-year prediction was 0.696, and the 3-year prediction was 0.898, indicating that the model had good applicability and better prediction of long-term prognosis.

### Effect of the mitophagy score on immune infiltration

**Figure 8:**
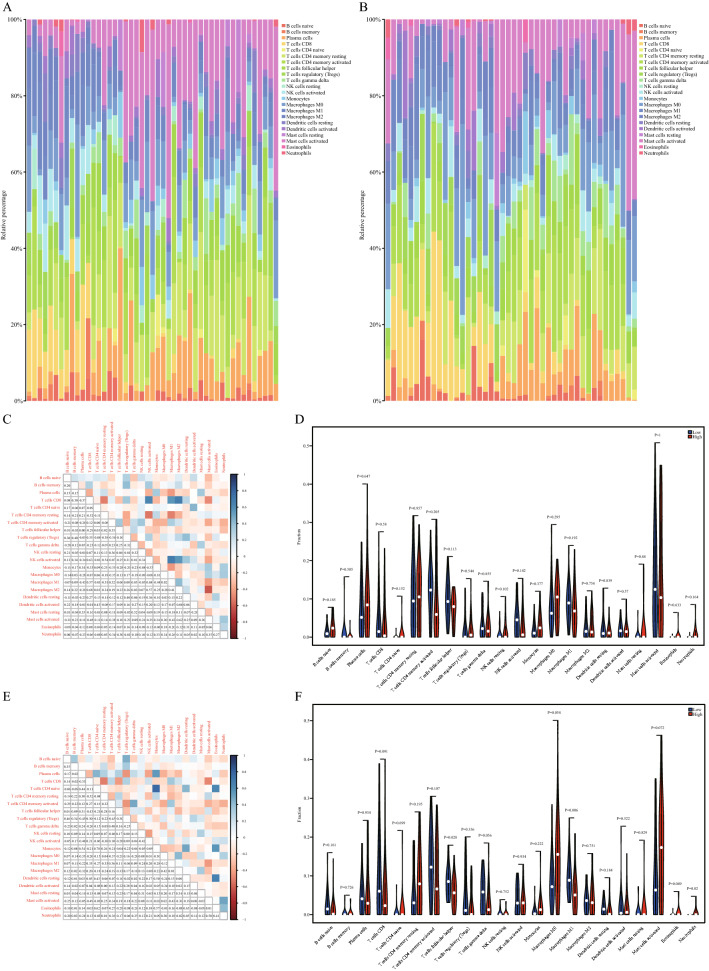
Effect of the mitophagy score on immune infiltration. (**A**) The CIBERSORT algorithm was used to analyse the training set, and then the immune cell correlation shown in the bar graph was obtained. (**B**) The CIBERSORT algorithm was used to analyse the validation set, and the immune cell correlation shown in the bar graph was obtained. (**A**–**B**) Software: R (4.0.2) version. R packet: ggplot2 [3.4.2]. URL: https://ggplot2.tidyverse.org; https://github.com/tidyverse/ggplot2). (**C**) Correlation of immune cells in the training set. D.The expression of immune cells in the training set according to mitophagy scoring grouping. (**C**, **E**) Software: R (4.0.2) version. R packet: corr plot [0.92]. URL: https://github.com/taiyun/corrplot). (**E**) Correlation of immune cells in the validation set. F.The expression of immune cells in the validation set according to mitophagy scoring grouping. (**D**, **F**) Software: R (4.0.2) version. R packet: ggplot2 [3.4.2]. URL: https://ggplot2.tidyverse.org; https://github.com/tidyverse/ggplot2).

To analyse the difference between the mitophagy regulation score model and the immune infiltration degree in the training set and the validation set, we calculated the infiltration degree of 22 immune cells in the training set and validation set. After removing the cells with total immune abundance values of 0, we analyse and include 15 kinds of immune cells, they were B cells naive, B cells memory, Plasma cells, T cells CD8, T cells CD4 memory resting, T cells follicular helper, T cells regulatory (Tregs), NK cells resting, NK cells activated, Monocytes, Macrophages M0, Macrophages M2, Dendritic cells activated, Mast cells resting, Mast cells activated (Fig. 8A–B).

To evaluate the functional correlation between key genes and immune cells in HPV-related HNSCC, we analysed the correlation between immune cells in the dataset (Fig. 8C–E) and the expression of immune cells in HPV-related HNSCC samples (Fig. 8D–F). The results showed that CD4^+^ memory T-cell and M2 macrophage infiltration decreased in the high-score group.

### Effect of Mitophagy score on prognosis

**Figure 9:**
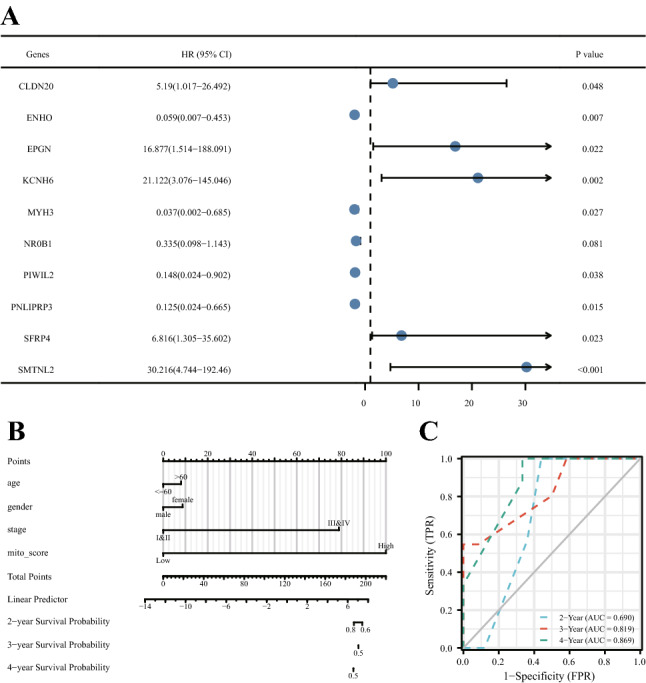
Effect of the mitophagy score on prognosis. (**A**) The forest map shows the multivariate Cox regression results of differentially expressed genes obtained by mitophagy regulatory gene subtypes. The first column represents the different genes, the second column represents the HR value and the corresponding 95% confidence interval, the fourth column shows the main part of the forest map (line segments, points, guides), and the fifth column represents the *P* value. (**B**) Nomogram of the correlation of the mitophagy regulatory score in the training set. (**C**) The ROC curve relies on time and is related to the risk score. The horizontal coordinate is the survival probability predicted by the model, the ordinate is the actual observed probability of survival.

Finally, the model was evaluated for prognostic prediction. First, multivariate Cox regression analysis was performed on the differentially expressed genes obtained by mitophagy regulation genotyping. Some of the results are shown in the forest map (Fig. 9A), indicating that gene expression had a significant impact on prognosis. Then, a nomogram was drawn for the training set based on the mitophagy regulation score (Fig. 9B), and it was found that the score could better predict the prognosis of patients. Cox analysis was conducted on the factors included in the nomogram to obtain the risk score, and an ROC curve was drawn at the same time (Fig. 9C). The results showed that the prediction efficiency at 2, 3 and 4 years was good, and the prediction efficiency of the model for long-term prognosis was more accurate, with the AUC value at 4 years reaching 0.869.

## Discussion

The incidence of HPV-related HNSCC is increasing yearly. Current studies have shown that the prognosis of HPV-related HNSCC is better than that of other head and neck tumours^29^, but there is no clear reason for this at present. Mitophagy is an important cellular process in the growth and apoptosis of normal cells. During the life cycle of mitochondria, gene expression or signalling pathways are changed due to inevitable harmful stimuli. To maintain the function of mitochondria in those cells, damaged mitochondria undergo a biological process called mitophagy that removes damaged mitochondria from the cell and maintains high-quality mitochondria in the cell. We speculated that there is a relationship between mitophagy and a favourable prognosis for HPV-related HNSCC. By searching for mitophagy-related DEGs in HPV-related HNSCC, the HPV-related HNSCC mitophagy score model was constructed, and the score model was verified. It was concluded that the constructed model can predict the prognosis of HPV-related HNSCC patients relatively accurately. However, we have not found a direct relationship between mitophagy and HPV-related HNSCC, and there is no relevant explanation for how the biological process of linear autophagy affects the progression of HPV-related HNSCC.

The mitophagy process is subject to the combined effects of various genes and related pathways. We used the mitophagy regulatory genes between HPV-related HNSCC and non-HPV-related HNSCC to conduct subtype studies, obtain the differentially expressed mitophagy-related genes, and showed the hub genes involved. Due to space limitations, we did not show all the genes, but the genes we showed were those that we thought were highly correlated with mitophagy during the analysis process. In addition, relevant basic experiments were performed to indirectly verify the reliability of the hub genes. For our experiments, the following representative differentially expressed genes were selected: NOS2, IL17REL, TMSB15A and TUBB4A. The results are consistent with our database analysis results. Because we did not collect enough HPV-related HNSCC specimens during the experiment, we used non-HPV-related HNSCC specimens and normal head and neck tissue specimens, which could be another reason for the uncertainty of the results. Next, we analysed HPV-related HNSCC samples to obtain HNSCC-related mitophagy DEGs and constructed an HPV-related HNSCC mitophagy score model based on the differentially expressed genes. In the process of constructing the score model, the mitophagy-related differentially expressed genes we selected were random, which may also be a limitation of our model. We also conducted correlation analysis on the immune infiltration of HPV-related HNSCC tissue in the high-score and low-score groups based on the mitophagy score model, and the results showed that the levels of CD4-positive memory T cells and M2 macrophages decreased in the high-score group. This suggests that the infiltration of CD4-positive memory T cells and M2 macrophages may lead to a better prognosis for HPV-related HNSCC. Although the expression of CD4-positive memory T cells can be significantly distinguished in our analysis, the P value at the time of analysis did not reach the threshold for statistical significance, but we believe that this may become a new research direction. Finally, we verified the score model, and the results showed that our score model could predict the prognosis of patients with HPV-related HNSCC to a certain extent.

Related studies have shown that combined melatonin and rapamycin treatment of HNSCC can induce changes in mitochondrial function^13^, which may be related to increased production of reactive oxygen species (ROS) in cells or mitochondria, increased apoptosis, or enhanced mitophagy. However, this study targeted all HNSCCs and did not distinguish HPV-related HNSCC from non-HPV-related HNSCC. Therefore, whether this study on mitophagy enhancement of HNSCC induced by tumour drugs can be applied to HPV-related HNSCC remains to be verified. Our study identified DEGs related to mitophagy in HPV-associated HNSCC. How these genes affect mitophagy and how they further affect the development of HPV-related HNSCC requires further research. Moreover, through the construction of a mitophagy score model, we characterized the infiltration of immune cells in HPV-related HNSCC, which initially showed a low level of CD4-positive memory T cells and M2 macrophages in the low-score group. We believe that this is meaningful and may aid in in the future development of immunotherapy against HPV-related HNSCC. At present, there are very few studies on mitophagy in HPV-related HNSCC, so the directions for future research are unclear. We hope that our study can provide aid in future HPV-related HNSCC research.

## Conclusion

In general, we investigated the link between mitophagy and HPV-related HNSCC by analysing DEGs. We believe that mitophagy can affect the prognosis of HPV-related HNSCC patients and propose that the high level of HPV expression may provide guidance for the development of precision medicine for HPV-related cancer patients.

## Supplementary Information

Supplementary Information.

## Data Availability

Data are available upon reasonable request from Dr. Liang Hui; e-mail:onlinelh@163.com.
