# Supplementary material for: Comprehensive analysis of mitophagy in HPV-related head and neck squamous cell carcinoma
Source: Sci Rep. 2023 May 9;13:7480. doi: 10.1038/s41598-023-34698-4 (PMC10170109; doi:10.1038/s41598-023-34698-4)
Supplement: Supplementary file 1 — Supplementary Information. [file 41598_2023_34698_MOESM1_ESM.docx]

**Supplementary Table1 Baseline data table of HPV-related TCGA-HNSCC**

|  | Overall | HPV_Negative | HPV_Positive | p |
| --- | --- | --- | --- | --- |
| n | 103 | 72 | 31 |  |
| age (mean (SD)) | 58.40 (11.02) | 59.11 (12.36) | 56.75 (6.88) | 0.321 |
| gender = female/male (%) | 20/83 (19.4/80.6) | 17/55 (23.6/76.4) | 3/28 (9.7/90.3) | 0.171 |
| T_stage (%) |  |  |  | 0.004 |
| NA | 18 (17.5) | 7 (9.7) | 11 (35.5) |  |
| 1 | 12 (11.7) | 9 (12.5) | 3 (9.7) |  |
| 2 | 22 (21.4) | 12 (16.7) | 10 (32.3) |  |
| 3 | 22 (21.4) | 17 (23.6) | 5 (16.1) |  |
| 4 | 1 (1.0) | 1 (1.4) | 0 (0.0) |  |
| 4a | 27 (26.2) | 25 (34.7) | 2 (6.5) |  |
| T0 | 1 (1.0) | 1 (1.4) | 0 (0.0) |  |
| N_stage (%) |  |  |  | 0.001 |
| NA | 19 (18.4) | 7 (9.7) | 12 (38.7) |  |
| 0 | 20 (19.4) | 17 (23.6) | 3 (9.7) |  |
| 1 | 14 (13.6) | 12 (16.7) | 2 (6.5) |  |
| 2 | 2 (1.9) | 1 (1.4) | 1 (3.2) |  |
| 2a | 2 (1.9) | 0 (0.0) | 2 (6.5) |  |
| 2b | 29 (28.2) | 19 (26.4) | 10 (32.3) |  |
| 2c | 15 (14.6) | 14 (19.4) | 1 (3.2) |  |
| N3 | 2 (1.9) | 2 (2.8) | 0 (0.0) |  |
| Stage (%) |  |  |  | 0.016 |
| NA | 21 (20.4) | 8 (11.1) | 13 (41.9) |  |
| I | 6 (5.8) | 5 (6.9) | 1 (3.2) |  |
| II | 9 (8.7) | 6 (8.3) | 3 (9.7) |  |
| III | 12 (11.7) | 9 (12.5) | 3 (9.7) |  |
| IVA | 53 (51.5) | 42 (58.3) | 11 (35.5) |  |
| IVB | 2 (1.9) | 2 (2.8) | 0 (0.0) |  |

**Supplementary Table2 Baseline data table of HPV-related GSE65858**

|  | Overall | HPV_Negative | HPV_Positive | p |
| --- | --- | --- | --- | --- |
| n | 256 | 196 | 60 |  |
| age (mean (SD)) | 60.20 (10.43) | 60.11 (10.85) | 60.49 (9.02) | 0.807 |
| gender = female/male (%) | 43/213 (16.8/83.2) | 29/167 (14.8/85.2) | 14/46 (23.3/76.7) | 0.177 |
| T_stage (%) |  |  |  | 0.053 |
| 1 | 35 (13.7) | 25 (12.8) | 10 (16.7) |  |
| 2 | 75 (29.3) | 53 (27.0) | 22 (36.7) |  |
| 3 | 56 (21.9) | 46 (23.5) | 10 (16.7) |  |
| 4a | 83 (32.4) | 69 (35.2) | 14 (23.3) |  |
| 4b | 7 (2.7) | 3 (1.5) | 4 (6.7) |  |
| N_stage (%) |  |  |  | 0.025 |
| 0 | 92 (35.9) | 81 (41.3) | 11 (18.3) |  |
| 1 | 30 (11.7) | 24 (12.2) | 6 (10.0) |  |
| 2a | 10 (3.9) | 7 (3.6) | 3 (5.0) |  |
| 2b | 62 (24.2) | 41 (20.9) | 21 (35.0) |  |
| 2c | 50 (19.5) | 35 (17.9) | 15 (25.0) |  |
| 3 | 12 (4.7) | 8 (4.1) | 4 (6.7) |  |
| Stage (%) |  |  |  | 0.199 |
| I | 18 (7.0) | 16 (8.2) | 2 (3.3) |  |
| II | 37 (14.5) | 31 (15.8) | 6 (10.0) |  |
| III | 35 (13.7) | 28 (14.3) | 7 (11.7) |  |
| IVA | 143 (55.9) | 106 (54.1) | 37 (61.7) |  |
| IVB | 16 (6.2) | 9 (4.6) | 7 (11.7) |  |
| IVC | 7 (2.7) | 6 (3.1) | 1 (1.7) |  |

**Supplementary Table3 GO enrichment analysis of DEGs**

| Ontology | Description | P.adjust |
| --- | --- | --- |
| BP | muscle system process | 1.67E-31 |
| BP | muscle contraction | 5.18E-31 |
| BP | keratinization | 9.42E-24 |
| BP | striated muscle contraction | 5.97E-23 |
| BP | myofibril assembly | 6.35E-23 |
| BP | striated muscle cell development | 8.32E-23 |
| BP | muscle cell development | 9.14E-23 |
| BP | muscle organ development | 8.76E-20 |
| BP | striated muscle tissue development | 6.14E-19 |
| BP | striated muscle cell differentiation | 2.08E-18 |
| CC | sarcomere | 1.56E-43 |
| CC | contractile fiber | 3.17E-43 |
| CC | myofibril | 5.35E-43 |
| CC | I band | 4.46E-25 |
| CC | Z disc | 5.46E-19 |
| CC | cornified envelope | 1.02E-13 |
| CC | sarcoplasmic reticulum membrane | 2.53E-10 |
| CC | myofilament | 7.87E-10 |
| CC | A band | 2.39E-09 |
| CC | muscle myosin complex | 2.68E-09 |
| MF | structural constituent of muscle | 1.77E-14 |
| MF | actin binding | 1.24E-13 |
| MF | actin filament binding | 1.81E-06 |
| MF | peptidase inhibitor activity | 2.18E-05 |
| MF | structural constituent of skin epidermis | 2.61E-05 |
| MF | FATZ binding | 6.76E-05 |
| MF | microfilament motor activity | 0.000144764 |
| MF | endopeptidase inhibitor activity | 0.000177353 |
| MF | peptidase regulator activity | 0.000177353 |
| MF | serine-type endopeptidase inhibitor activity | 0.000183531 |

**Supplementary Table4 KEGG enrichment analysis of DEGs**

| ID | Description | p.adjust |
| --- | --- | --- |
| hsa04260 | Cardiac muscle contraction | 3.31E-08 |
| hsa04261 | Adrenergic signaling in cardiomyocytes | 4.07E-07 |
| hsa05414 | Dilated cardiomyopathy | 4.63E-07 |
| hsa05410 | Hypertrophic cardiomyopathy | 1.63E-06 |
| hsa04020 | Calcium signaling pathway | 4.36E-06 |
| hsa05412 | Arrhythmogenic right ventricular cardiomyopathy | 0.001549095 |
| hsa05150 | Staphylococcus aureus infection | 0.006389447 |
| hsa04022 | cGMP-PKG signaling pathway | 0.01382538 |

**Supplementary Table5** **GSEA analyze**

| Description | enrichmentScore | p.adjust |
| --- | --- | --- |
| KEGG_RIBOSOME | 0.654254876 | 1.05E-08 |
| KEGG_FOCAL_ADHESION | -0.562103898 | 1.05E-08 |
| KEGG_SPLICEOSOME | 0.53721418 | 1.05E-08 |
| KEGG_DNA_REPLICATION | 0.757616864 | 2.11E-08 |
| KEGG_HYPERTROPHIC_CARDIOMYOPATHY_HCM | -0.642214912 | 7.11E-07 |
| KEGG_ECM_RECEPTOR_INTERACTION | -0.637674264 | 8.57E-07 |
| KEGG_CELL_CYCLE | 0.492010672 | 9.42E-07 |
| KEGG_CYTOKINE_CYTOKINE_RECEPTOR_INTERACTION | -0.50325216 | 9.42E-07 |
| KEGG_DILATED_CARDIOMYOPATHY | -0.620292722 | 2.88E-06 |
| KEGG_HUNTINGTONS_DISEASE | 0.420840055 | 6.49E-06 |
| HALLMARK_E2F_TARGETS | 0.687918477 | 8.33E-10 |
| HALLMARK_EPITHELIAL_MESENCHYMAL_TRANSITION | -0.651300505 | 8.33E-10 |
| HALLMARK_G2M_CHECKPOINT | 0.597428518 | 8.33E-10 |
| HALLMARK_MYC_TARGETS_V1 | 0.552443414 | 8.33E-10 |
| HALLMARK_MYOGENESIS | -0.735900431 | 8.33E-10 |
| HALLMARK_OXIDATIVE_PHOSPHORYLATION | 0.499110284 | 8.33E-10 |
| HALLMARK_INFLAMMATORY_RESPONSE | -0.552695192 | 5.79E-09 |
| HALLMARK_KRAS_SIGNALING_UP | -0.52995058 | 2.11E-07 |
| HALLMARK_COAGULATION | -0.553669064 | 5.23E-06 |
| HALLMARK_MYC_TARGETS_V2 | 0.578684858 | 1.17E-05 |

**Supplementary Table6**  **GSVA analyze**

| id | logFC | adj.P.Val |
| --- | --- | --- |
| KEGG_OLFACTORY_TRANSDUCTION | 0.157276998 | 1.31E-16 |
| KEGG_HOMOLOGOUS_RECOMBINATION | -0.199077777 | 1.01E-05 |
| KEGG_NUCLEOTIDE_EXCISION_REPAIR | -0.163296268 | 1.42E-05 |
| KEGG_MISMATCH_REPAIR | -0.206420475 | 3.24E-05 |
| KEGG_PPAR_SIGNALING_PATHWAY | 0.089881563 | 0.000112281 |
| KEGG_CELL_CYCLE | -0.128032895 | 0.000155925 |
| KEGG_DNA_REPLICATION | -0.208448615 | 0.000363179 |
| KEGG_NEUROACTIVE_LIGAND_RECEPTOR_INTERACTION | 0.098736655 | 0.000578726 |
| KEGG_SPLICEOSOME | -0.149446279 | 0.002027715 |
| KEGG_BASE_EXCISION_REPAIR | -0.164899609 | 0.002270985 |
| HALLMARK_PANCREAS_BETA_CELLS | 0.103123835 | 6.62E-05 |
| HALLMARK_KRAS_SIGNALING_DN | 0.081686406 | 0.000114076 |
| HALLMARK_E2F_TARGETS | -0.177628214 | 0.000444683 |
| HALLMARK_DNA_REPAIR | -0.113872471 | 0.00071067 |
| HALLMARK_MYC_TARGETS_V1 | -0.139940428 | 0.002455577 |
| HALLMARK_G2M_CHECKPOINT | -0.135144575 | 0.004660571 |
| HALLMARK_COAGULATION | 0.102347229 | 0.006135321 |
| HALLMARK_OXIDATIVE_PHOSPHORYLATION | -0.121951595 | 0.015889802 |
| HALLMARK_MTORC1_SIGNALING | -0.073445113 | 0.062035085 |
| HALLMARK_MYC_TARGETS_V2 | -0.126707089 | 0.087206216 |
